# Influence of Soil Depth and Land Use Type on the Diversity of and Metabolic Restriction in the Soil Microbial Community of a Forest-Grass Ecotone

**DOI:** 10.3390/microorganisms13071450

**Published:** 2025-06-22

**Authors:** Xuman Ma, Xiaogang Li, Yaxin Meng, Jinhua Liu, Jinxin Wang, Xiaomeng Yu, Weipeng Wang, Xuehua Xu

**Affiliations:** College of Forestry, Hebei Agricultural University, Baoding 071001, China; m150326696871@163.com (X.M.); xiaogang1217@126.com (X.L.); 17692708355@163.com (Y.M.); 15227630356@163.com (J.L.); 15233302800@163.com (J.W.); 15075188793@163.com (X.Y.); 15564992527@163.com (W.W.)

**Keywords:** forest-grass ecotone, land use types, microbial metabolic restriction, soil microbial diversity, soil depth

## Abstract

Revealing soil microbial diversity and metabolic limitations in different land uses and soil depths is essential to understanding the regulation processes of soil nutrients. Here, bacterial and fungal microbial diversity and metabolic restriction in the 0–50 cm soil layers of four land uses, namely farmland, grassland, *Betula platyphylla* secondary forest, and *Larix principis-rupprechtii*-planted forest in the mountainous forest-grass ecotone of northern China, were determined. The results showed that soil microbial diversity in farmland was the lowest. Soil microorganisms from all land uses are limited by nitrogen, with the highest nitrogen limitation in planted forest. However, microbial nitrogen limitation in farmland increased with increasing soil depth, while microbial nitrogen limitation in grassland, secondary forest, and planted forest decreased with increasing soil depth. The bacterial and fungal community composition was influenced by soil organic carbon, total nitrogen, soil organic carbon:total phosphorus ratio, soil water content, soil organic carbon, and total nitrogen:total phosphorus ratio. The soil organic carbon:total phosphorus ratio has an impact on microbial metabolic limitation. This study shows that soil microbial communities were more affected by land-use type than soil depth. Land use changes the input of soil nutrients from aboveground plants, which affects the physical and chemical properties of soil, microbial community diversity, and microbial metabolic limitation. The vertical filtration effect between soil layers reduces soil nutrients, making the microbial diversity and enzyme activity of surface soil greater than those of deep soil. Our study helps to understand the function of soil microorganisms under different land use types in the forest-grass ecotone of northern China and provides a basis for predicting biogeochemical cycle dynamics in the ecotone in the context of global warming.

## 1. Introduction

Nutrient cycling in soil is facilitated by soil microbes, and an unbalanced nutrient supply in the soil will impact the metabolic state of these microorganisms [1]. By creating and secreting a variety of extracellular enzymes, soil microorganisms break down macromolecular organic compounds into smaller molecules. These enzymes are then used to obtain energy and nutrients for the growth of the microorganisms [2,3,4]. Because extracellular enzymes can respond to the availability of nutrients in the environment, it is believed that extracellular enzymes constitute a critical link between biological metabolism and biostoichiometry [5,6]. Soil carbon (C), nitrogen (N), and phosphorus (P) acquisition enzymes are divided according to the nutrient conversion function of extracellular enzymes [7]. The relative activity of these three types of enzymes, i.e., extracellular enzyme activity (EEA), is regulated by soil nutrients. As a result, EEA reflects the stoichiometric balance of microbial nutrient and resource demand [8]. In addition, EEA is often used to reflect changes in soil microbial cellular metabolism due to nitrogen deposition, land use change, nutrient addition, and climate change. It also illustrates the connection between the environment’s nutrient availability and the distribution of soil microbial resources [9,10,11,12]. Therefore, to quantify the microbial resource restrictions and the nutrient limitation of ecosystems, most studies have employed soil extracellular enzyme stoichiometry [13,14]. In order to shed more light on the metabolic characteristics of bacteria, Moorhrad et al. [15] employed vector length and angles to characterize C, N, and P limitations in the ecosystem. Cao et al. [5] used enzyme activity vector analysis to investigate the metabolic limitations of microbes in the rhizosphere soil of Quercus aquifolioides. In order to investigate the geographical distribution of microbial nutrient limitation in alpine meadows and desert regions, Sun et al. [16] employed vector analysis and enzyme stoichiometry.

Land use change represents a key driver of soil nutrient dynamics and subsequent shifts in soil microbial community structure [17]. Different land use types showed differences in the nutritional composition of the soil, which affects the relative abundance of soil microorganisms [18]. A study revealed that pH value, organic matter, and N, P, and potassium (K) contents significantly affect soil microbial communities [19]. Similar findings were discovered by Li et al. [20] regarding differences in bacterial community composition and diversity, as well as nutritional components, across various land use types. The varying concentrations of soil nutrients across different land use categories could be linked to the metabolic limitations faced by soil microbes, which stem from the restricted availability of these nutrients [21]. Consequently, this kind of research is very meaningful. In a study focused on Robinia pseudocacia plantations in hilly and gully regions of loess [22], N was observed to be the limiting factor for soil microbial metabolism. Similarly, it was discovered that both N and phosphorus hindered microbial metabolism in several desert types in northwest China [23]. Yao et al. [24] studied constraints on microbial metabolism in the forest and farmland of the hilly and gully region of the Loess Plateau. Some studies have found that restoration of forest vegetation is mainly hindered by phosphorus, while grassland and farmland are mainly limited by nitrogen. Natural grassland has the lowest microbial restriction of C and P, which is conducive to ecological restoration [4].

One important factor influencing nutrient conditions and soil microbial communities is environmental heterogeneity. Furthermore, soil microbial community composition, diversity, and metabolic limitations generally vary across spatial scales. In a recent study, spatial distribution patterns of soil microbial nutrient restriction in 31 ecosystems in China were studied. Due to variations in the spatial distribution of ecosystems, nearly half of the ecosystems showed differences in microbial nutrient limitations (mainly P limitation), exhibiting an important relationship between microbial nutrient restriction characteristics and soil pH [25]. According to Nottingham et al. [26], as altitude increased, microbial nutrient constraint changed from P limitation to N limitation. These results imply that geographical location affects microbial metabolic restriction. Consequently, in order to comprehend the soil nutrient status in tiny regions, it is crucial to carry out an exhaustive examination.

The soil’s nutrient status, as well as the microbial community’s structure, are influenced by soil depth. Understanding the connection between soil conditions and environmental variability has been hampered in the majority of studies to date, which have mostly concentrated on the nutrients and microbial characteristics of surface soil, while paying less attention to changes in deep soil [27]. Chu et al. [28] found that differences in bacterial communities at a soil depth of 30 cm remain almost unchanged compared with those in deeper soils. In a recent study, soil bacteria existing at a 2 m soil depth under different tree species exhibited greater depth sensitivity in the soil than fungal communities [29]. Depth-dependent patterns of microbial resource restrictions are caused by the depth of the soil. Research has indicated that, as soil depth increases, microbial P limitation increases as well [30]. However, the effect of soil depth on microbial nitrogen restriction is unclear, due to spatial heterogeneity. A thorough comprehension of metabolic limitation in the soil can be helpful in developing strategies for the rational use of land resources. Therefore, investigating microbial nutrient limitation in different soil layers of this region is crucial.

A forest-grass ecotone is an area where forest and grassland ecosystems are interleaved on a certain spatial and temporal scale. These areas have high species richness and high productivity. However, forest-grass ecotones are unstable, as their structure and function are easily affected by natural and human interferences [31]. The northern mountains of Hebei are an important ecological multi-functional area in the forest-grass ecotone of northern China. This region not only has a staggered forest-grass ecotone, but also has staggered areas of agriculture and animal farming. This region coexists with a variety of land use types, including farmland, grassland, and forest, and the nutrient status is complicated and variable. Previously, soil nutrients in the northern mountains of Hebei have been investigated by the researchers; however, soil microbial metabolic limitation has been rarely explored in this region. It is imperative to explore the variety and metabolic limits of soil microbial communities and identify potential influencing factors in order to lay down a scientific basis for land management in this region.

The goals of this study were to better understand soil microbial functions under different land use types in the forest-grass ecotone of northern China and to provide a fundamental basis for predicting the dynamics of biogeochemical cycles in this ecotone in the context of climate warming. For this research, we collected soil samples from farmland, grassland, secondary forest, and planted forest in northern Hebei Province. ITS and 16S rRNA high-throughput sequencing were used to examine the bacterial and fungus communities present in the soil. The objectives of our research were to determine (1) any differences in soil microbial community composition and diversity among different land use types and soil depth in the forest-grass ecotone of northern China, (2) if soil metabolic limitations vary with land use, and (3) what environmental factors most strongly influence microbial community structure, diversity, and nutrient limitation.

## 2. Materials and Methods

### 2.1. Study Sites and Sample Collection

**Figure 1:**
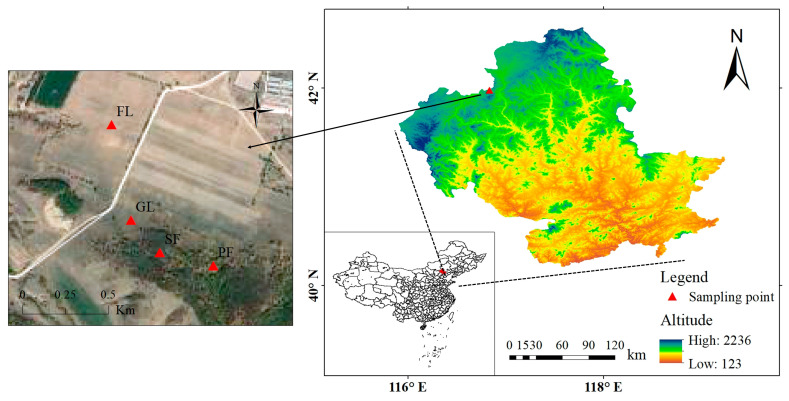
Location of the studied area.

The research site is situated in Laowopu Township, Weichang County, Chengde City, northern Hebei Province (42°2′7.439′′ N, 116°59′39.372′′ E). The average elevation of the site is 1021 m. The yearly average temperature is 5.1 °C, with 373 mm of precipitation on average. It is located in the boundary zone of the Inner Mongolia Plateau and North China, between the forested northern mountains of Hebei and the grasslands of Inner Mongolia. A geographical location map of the study area is shown in Figure 1.

From July to August 2023, four typical land use types (farmland, grassland, secondary forest, and planted forest) in the typical forest and grassland transition belt in Laowopu were investigated. The basic overview of the sample plot is shown in Table 1. The area was originally a secondary forest of *Betula platyphylla*. In the late 1970s, due to the living and production needs of the residents in this area, different parts were transformed into farmland, grassland, secondary forest, and planted forest.

There were three 20 × 30 m sample plots created for each type of land use. At each sample plot’s four corners and center, soil samples were collected from five distinct soil strata (0–10, 10–20, 20–30, 30–40, and 40–50 cm). A total of 60 samples were collected. These soil samples were manually cleaned by removing stones, litter, and roots. Subsequently, the soil samples were sieved through a 2 mm sieve. After collecting the soil samples, they were immediately stored at −80 °C to ensure the activity of soil microorganisms. Then, the microbial genomes were isolated and subjected to high-throughput sequencing. For soil physicochemical property analysis, a portion of each soil sample was kept in a polyethylene plastic bag and kept at room temperature.

### 2.2. Analysis of the Physical and Chemical Properties of Soil

Soil pH and soil water content (SWC) were determined by pH meter at a 2.5:1 soil to water ratio by the mass ratio method and ring knife method, respectively. Soil organic carbon (SOC): potassium dichromate external heating method; total nitrogen (TN): Kjeldahl method with sulfuric acid digestion; total phosphorus (TP): molybdenum-antimony colorimetry after sulfuric acid digestion [32].

### 2.3. Analysis of Microbial Metabolic Restriction

Microplate fluorescence quantification was used to quantify the extracellular enzyme activities of the soil. The C-acquiring enzymes are represented by β-1,4-glucosidase (BG) and β-D-cellobiose hydrolase (CBH); β-1,4-N-acetylglucosaminosidase (NAG) and leucine aminopeptidase (S-LAP) are examples of N-acquiring enzymes; and the P-acquiring enzyme is acid phosphatase (AP). Microbial C metabolic restriction was characterized using vector length [6]:

(1) $$Length=\sqrt{\left(x^{2}+y^{2}\right)}$$

The variables *x* and *y* represent the relative activity of enzymes acquiring C and N and C and P, respectively. The longer the vector, the higher the microorganism’s C limitation.

N and P metabolic limitations were characterized using vector angles:

(2) $$Angle\left({}^{\circ}\right)=DEGREES\left(A\;tan2\left(x,y\right)\right)$$

The vector angles of N and P were the cut-off for the line, where the drawing origin extends to the point (*x*, *y*). When the vector angle was less than 45°, microorganisms were constrained by N, and when it was more than 45°, by P. As the angle widened, the P limitation went up and the N limitation went down.

### 2.4. Extraction of Soil Microbial DNA and High-Throughput Sequencing

Using a DNA extraction kit (TiangenDP812), we first extracted the genomic DNA of soil microorganisms. The primers used for amplification of the V3-V4 regions of 16SrRNA of soil bacterial DNA were F: ACTCCTTACGGGAGGCAGCA and R: GGACTACHVGGGTWTCTAAT. The primers used for targeting the ITS 1-F regions of soil fungi were F: CCTGGTCATTTAGAGGAAGTAA and R: GCTGCGTTCTTCATCGATGC. To create qualified sequencing libraries, amplicons underwent purification, quantification, and homogenization. To obtain the raw sequences (reads), we used an Illumina NovaSeq 6000 platform for high-throughput sequencing. Trimmomatic v0.33 was used to filter these raw reads. To obtain clean reads, primer sequences were found and eliminated using Cutadapt 1.9.1. DADA 2 in QIIME2 2020.6 was used for denoising and clustering of sequences at 100% similarity to obtain amplicon sequence variants (ASVs). The SILVA and UNITE databases were utilized for annotation of related bacterial and fungal species, respectively.

### 2.5. Statistical Analyses

Based on the ASV classification results, the diversity and richness indices of bacteria and fungi (ACE, Chao1 richness index, Shannon–Wiener index, and Simpson index) were calculated. R (v.3.6.1) and SPSS (v.20.0) were used for statistical and differential analyses of bacterial and fungal diversity indices. The Bray–Curtis method was used to conduct PCoA analysis in order to evaluate differences in the soil microbial community structure at different soil depths and for different land use types. We employed one-way ANOVA to assess variations in soil extracellular enzyme activities across different land use types and within various soil layers. SPSS (v.20.0) was used for plotting.

Spearman correlation analysis was employed to assess the relationships among the α diversity of soil microorganisms, environmental factors, and extracellular enzyme-related indices. Additionally, a Mantel test was conducted and visualized using the R “vegan” package. Redundancy analysis (RDA) was further utilized to explore these relationships in greater depth, with visualization performed using Canoco 5.0 software.

## 3. Results

### 3.1. Basic Physical and Chemical Properties of Soil

**Figure 2:**
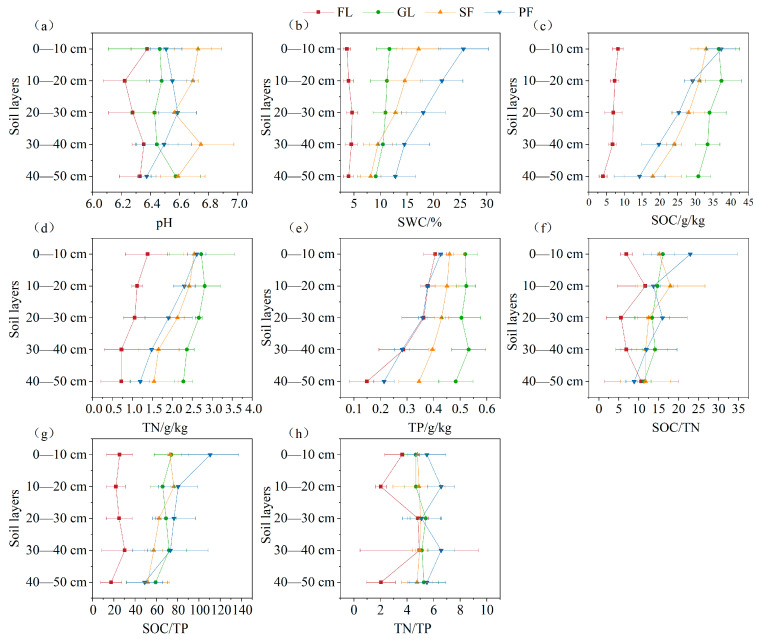
Soil physical and chemical properties under different land use types and at different soil depths. Note: (**a**) pH content (**b**) Soil water content; (**c**) Organic carbon content; (**d**) Total nitrogen content; (**e**) Total phosphorus content; (**f**) Organic carbon: Total nitrogen content; (**g**) Organic carbon: Total phosphorus content; (**h**) Total nitrogen: Total phosphorus content. FL: farmland, GL: grassland, SF: secondary forest, PF: planted forest. The same below.

The soil pH for the four land use types was weakly acidic (Figure 2). The soil water content (SWC) for planted forest was the highest, the SWC for farmland was the lowest, and the SWC gradually decreased with increasing soil depth. The contents of soil organic carbon (SOC), total nitrogen (TN), and total phosphorus (TP) in the four land use types were grassland > secondary forest > planted forest > farmland and showed a decreasing trend with increasing soil depth. The SOC/TN value for grassland, secondary forest, and planted forest decreased with increasing soil depth, while the SOC/TN value for farmland increased first, then decreased, then increased. The SOC/TP value showed a decreasing trend among soil layers of the four land use types.

### 3.2. Composition and Diversity of Soil Bacterial and Fungal Communities

**Figure 3:**
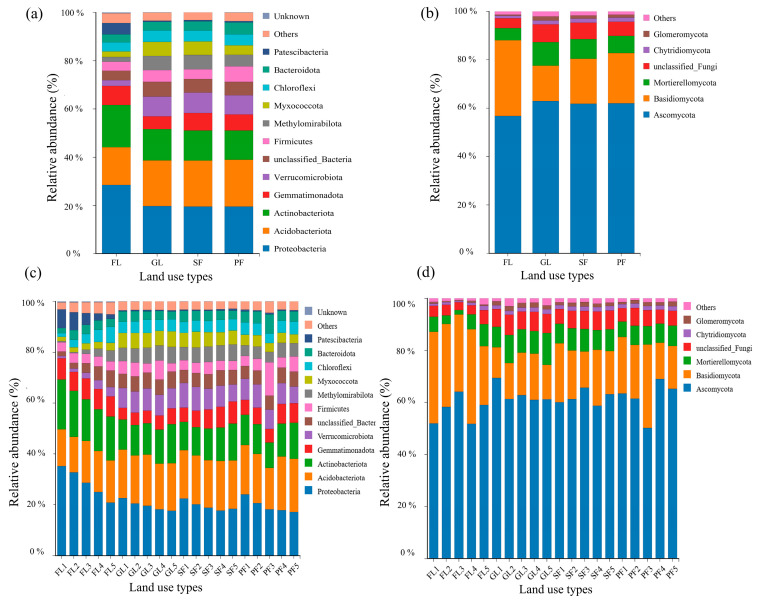
Composition of soil bacterial and fungal communities for different land use types and soil depths. Note: (**a**) Relative abundances at the bacterial phylum level under different land use types; (**b**) Relative abundances at the fungal phylum level under different land use types; (**c**) Relative abundances at the bacterial phylum level under different soil depths; (**d**) Relative abundances at the fungal phylum level under different soil depths.

Following high-throughput sequencing, soil samples from four different land use types contained 24,910 fungal ASVs and 106,988 bacterial ASVs. At the phylum level, 14 dominant bacterial phyla with relative abundance >1% and 7 dominant fungal phyla were detected. Bacterial taxa with relative abundance <1% were classified as “Other” (Figure 3a). For the four land use types, the abundances of Proteobacteria, Acidobacteriota, and Actinobacteriota were relatively high. The highest abundances of Proteobacteria (28.57%) and Actinobacteria (17.56%) were observed in farmland soil, while the abundance of Acidobacteriota (19.40%) was highest in planted forest soil. Similarly, Ascomycota and Basidiomycota were the most dominant fungal phyla (Figure 3b). The highest abundance of Ascomycota was observed in grassland soil, while the highest abundance of Basidiomycota was witnessed in farmland soil.

In each soil layer, Proteobacteria, Acidobacteriota, and Actinobacteria were observed to be the top three dominant bacterial phyla with relative abundance >1%. For all four land use types, the abundance of Proteobacteria decreased with soil depth (Figure 3c). Similarly, Ascomycota and Basidiomycota showed the highest abundance in each soil layer. However, no clear pattern of change in abundance was observed with soil depth for the four land use types (Figure 3d).

**Figure 4:**
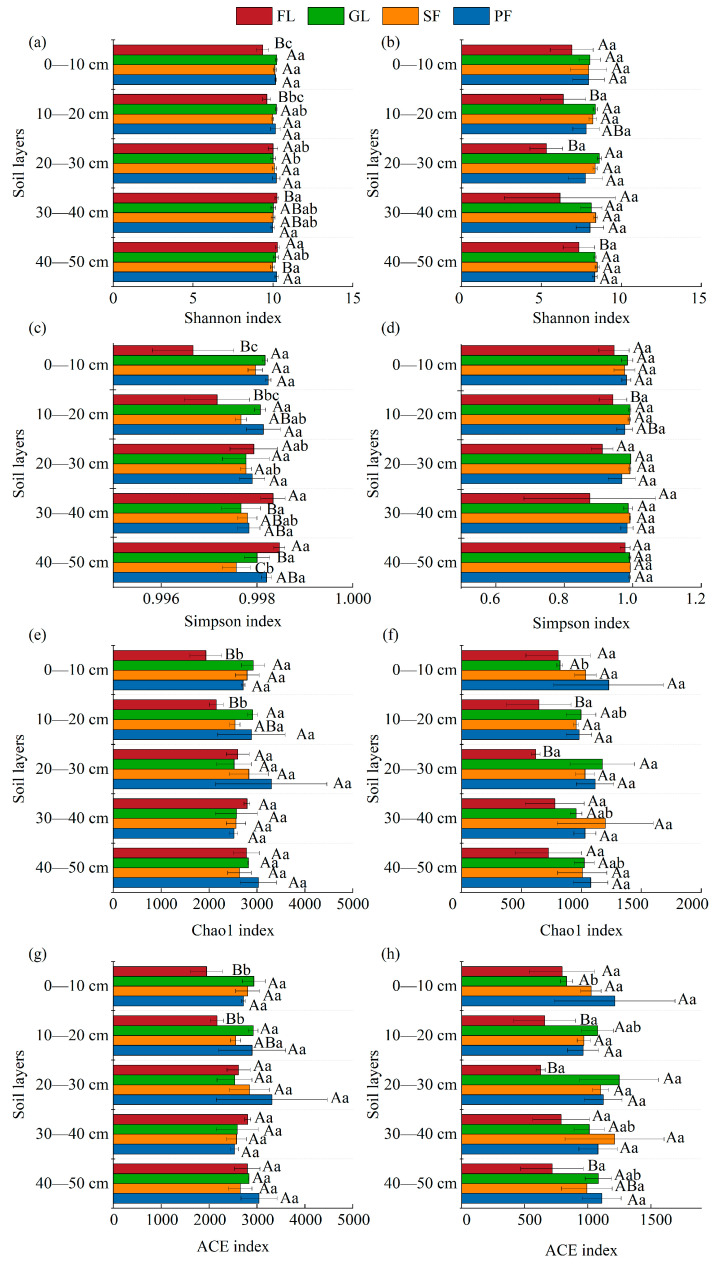
Shannon index (**a**,**b**), Simpson index (**c**,**d**), Chao1 index (**e**,**f**), and ACE index (**g**,**h**) of bacteria and fungi under different land use patterns. Note: Different uppercase letters indicate statistically significant differences among different land use types (*p* < 0.05), while different lowercase letters indicate statistically significant differences among different soil depths (*p* < 0.05).

The Shannon index and Simpson index of bacteria and fungi showed basically no significant differences in the different soil layers, and the Chao1 index and ACE index also exhibited the same results (Figure 4). Bacteria had greater species diversity and richness than fungi. Farmland had the lowest levels of fungal species diversity and richness. The diversity and abundance of bacteria in the surface soil layers (0–10 cm, 10–20 cm) of farmland are usually significantly lower than those for other land use types (*p* < 0.05).

**Figure 5:**
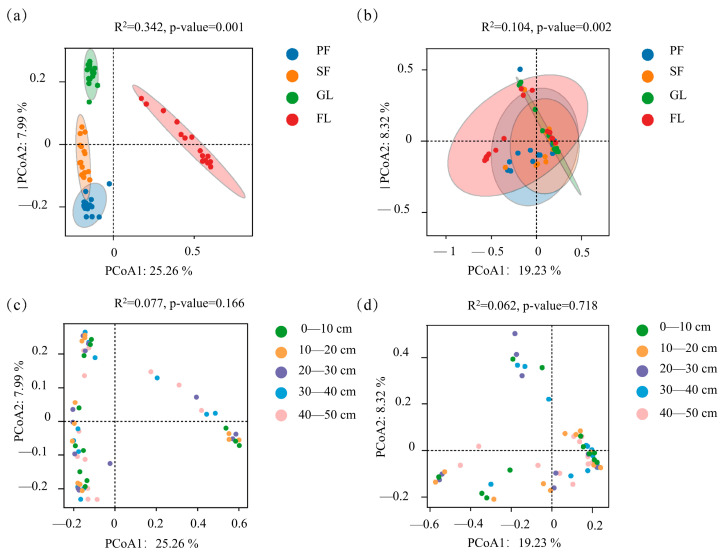
Results of the PCoA analysis based on a Bray–Curtis heterogeneity matrix, representing changes in bacterial (**a**,**c**) and fungal (**b**,**d**) communities for different land use types and at different soil depths.

PCoA of the ASVs of bacteria and fungi for different land use types and different soil layers was performed (Figure 5). Regarding bacterial β diversity, the first axis contributed 25.26%, while the second axis contributed 7.99%, with the two axes explaining 33.25% of the variance. Regarding the fungal community, the first axis contributed 19.23%, while the second axis contributed 8.32%, with the two axes explaining 27.55% of variance. Both bacterial and fungal communities were significantly affected by land use type (*p* < 0.01) (Figure 5a,b). On the first coordinate axis, the distance between farmland and the other three land use types is large. This indicated a significant difference between the bacterial community structures of farmland and the other three land use types. The distribution of samples in each group of fungal communities was relatively clustered, and the similarity of the fungal communities was high. Figure 5c,d show that the bacterial community structure in each soil layer is similar, and the soil layer has no significant effect on bacterial and fungal community structure.

### 3.3. Stoichiometric Changes and Metabolic Limitations of Microbial Enzymes in Soil

**Figure 6:**
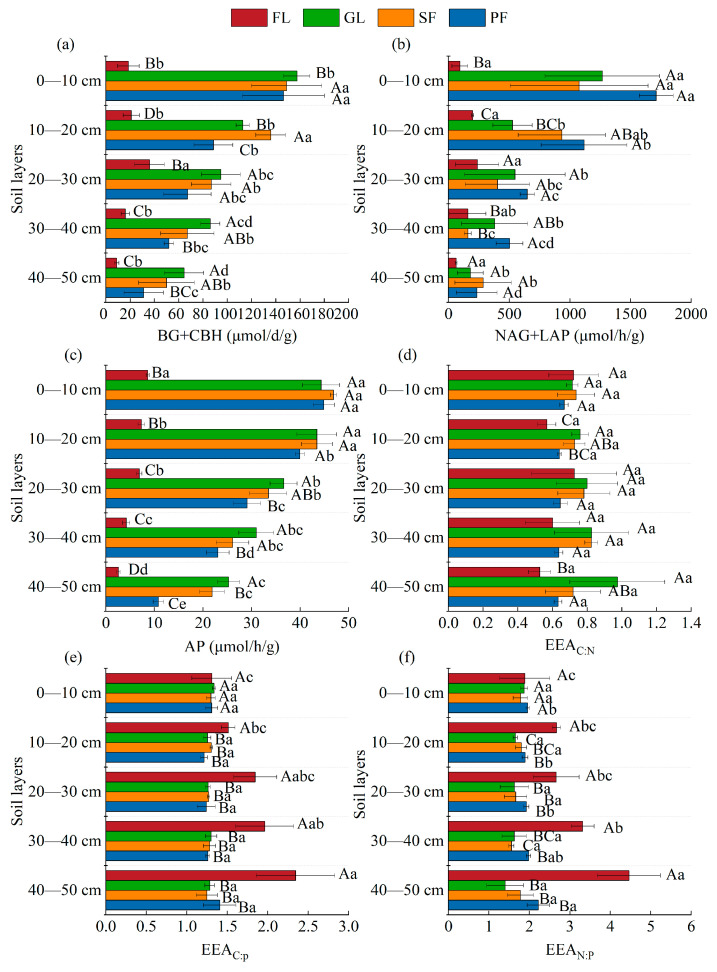
Effects of different land use types and soil depth on soil extracellular enzyme activities and their stoichiometry in soil. Note: (**a**) C-acquiring enzymes content; (**b**) N-acquiring enzymes content; (**c**) P-acquiring enzymes content; (**d**) The activity ratio of C and N enzymes; (**e**) The activity ratio of C and P enzymes; (**f**) The activity ratio of N and P enzymes. BG, β-1, 4-glucosidase (μmol·d^−1^·g^−1^); CBH, β-D-cellosidase (μmol·d^−1^·g^−1^); NAG, β-1, 4-N-acetylglucosaminidase (μmol·h^−1^·g^−1^); LAP, L leucine aminopeptidase (μmol·h^−1^·g^−1^); AP, alkaline phosphatase (μmol·h^−1^·g^−1^); EEA_C:N_-enzyme: LN[(BG + CBH)/(NAG + LAP)], EEA_C:P_ enzyme, LN[(BG + CBH)/AP]; EEA_N:P_-enzyme, LN[(NAG + LAP)/AP]. Different uppercase letters indicate statistically significant differences among different land use types (*p* < 0.05), while different lowercase letters indicate statistically significant differences among different soil depths (*p* < 0.05).

Except for farmland, the activities of C-, N-, and P-related enzymes in the surface soil of the other land use types were higher than those in deep soil, and the activities of three enzymes in farmland were the lowest for all land use types (*p* < 0.05) (Figure 6). Farmland had the lowest value of the C-acquiring enzyme, whereas grassland had the highest value. Except for the 40–50 cm soil layer, the N-acquiring enzyme activity of planted forest was the highest among the four land use types. The P-acquiring enzyme activity in the 0–50 cm soil layer was substantially lower in farmland than it was in the other three land types.

The EEA_C:N_ of farmland and plantations was significantly lower than that of grassland in the 10–20 cm soil layer (*p* < 0.05). The EEA_C:P_ and EEA_N:P_ of farmland were significantly higher than other land use types, except for 0–10 cm soil layer. The EEA_C:P_ of the 40–50 cm soil layer for farmland was significantly higher than that of the 0–10 cm and 10–20 cm soil layers (*p* < 0.05). The EEA_N:P_ for the 40–50 cm soil layer of farmland was significantly higher than that for the other soil layers (*p* < 0.05).

**Figure 7:**
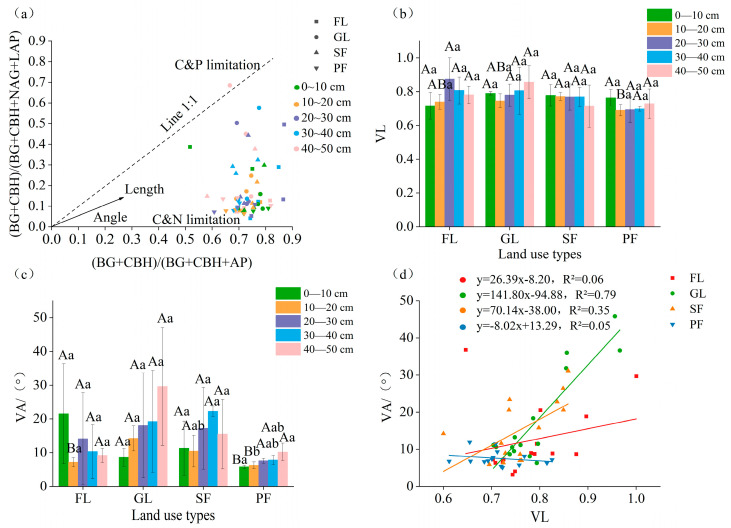
Enzyme stoichiometry (**a**), vector lengths (**b**), vector angles (**c**), and linear regression fitting (**d**) of different land use types and for different soil layers. Note: VL: vector length; VA: the vector angle. Different uppercase letters indicate statistically significant differences among different land use types (*p* < 0.05), while different lowercase letters indicate statistically significant differences among different soil depths (*p* < 0.05).

The nitrogen restriction on microbial metabolism for the four land use types is explained by the position of most data points below the 1:1 line (Figure 7a). In the 10–20 cm soil layer, the vector length for secondary forest was significantly higher than that for planted forest (Figure 7b). The vector angle for the 0–10 cm soil layer of planted forest was significantly lower than that for other land use types, and the vector angle for the 10–20 cm layer of farmland and planted forest was significantly lower than that of grassland and secondary forest (*p* < 0.05) The planted forest had a substantially smaller vector angle, but the grassland had the largest angle (Figure 7c). The surface soil of farmland had a higher vector angle than the deep soil, whereas the grassland, secondary forest, and planted forest had the opposite vector angles. Linear regression analysis revealed that the vector length and vector angle of the different land use types correlated positively (Figure 7d).

### 3.4. Correlation of Soil Properties, Microbial Communities, and Their Metabolic Constraints

**Figure 8:**
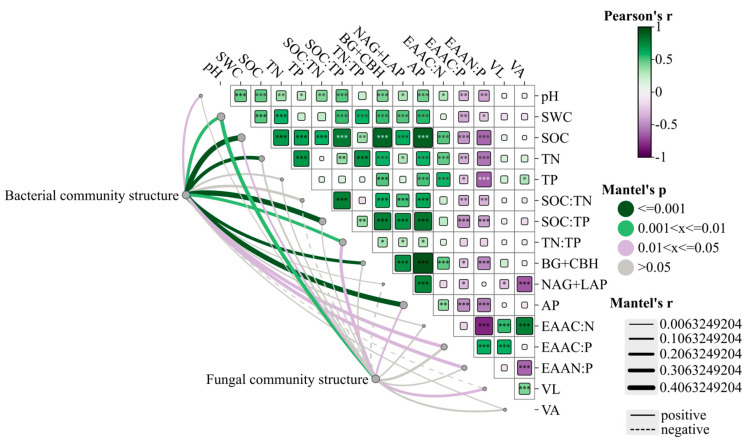
Spearman correlation analysis of soil physical and chemical properties and dominant community composition at the microbial phyla level. Note: *, **, *** indicated that the differences were statistically significant, *p* < 0.05, *p* < 0.01, *p* < 0.001.

Spearman correlation analysis showed that C-, N-, and P-acquiring enzymes and soil properties also showed significant correlations (Figure 8). C-, N-, and P-acquiring enzymes are closely related to each other. C-acquiring enzymes and EAA_N:P_ were found to be significantly and negatively correlated (*p* < 0.001). Similarly, P-acquiring enzymes showed very significant negative correlations with EAA_C:P_ and EAA_N:P_ (*p* < 0.001). EAAN:P was significantly negatively correlated with EAA_C:N_, and EAA_N:P_ was significantly positively correlated with EAA_C:P_ (*p* < 0.001). Vector length showed very significant positive correlations with EAA_C:N_ and EAA_C:P_ (*p* < 0.001). Vector angle and N-acquiring enzymes showed very significant negative correlations with EAA_N:P_ (*p* < 0.001) and significant positive correlations with EAA_C:N_ (*p* < 0.001). The Mantel test shows that the bacterial community composition was affected by soil SOC, TN, and SOC:TP and had a significant positive correlation with the activities of C- and P-acquiring enzymes. Furthermore, significant correlations of bacterial community composition were observed with EAA_C:P_ and EAA_N:P_ (*p* < 0.05). In addition, fungal community composition showed weak correlations with most indicators. Fungal community composition only showed very significant positive correlations with SWC (*p* < 0.01).

**Figure 9:**
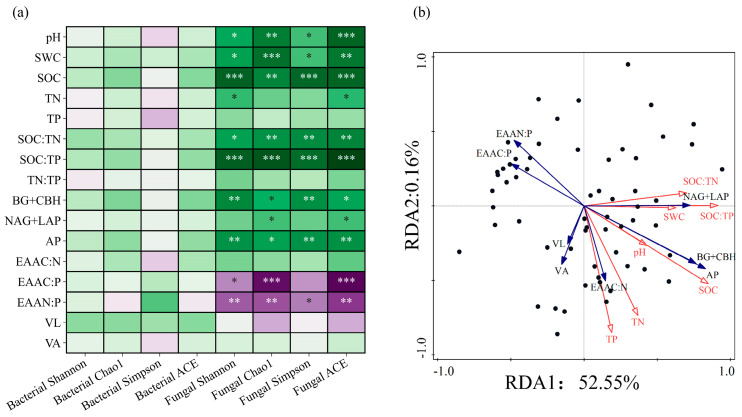
Spearman correlation analysis of soil physical and chemical properties and microbial diversity at the microbial phyla level (**a**), restriction of soil enzyme metabolism and redundancy analysis of physical and chemical factors (**b**). Note: *, **, *** indicated that the differences were statistically significant, *p* < 0.05, *p* < 0.01, *p* < 0.001.

No significant correlations of bacterial α diversity were observed with soil properties, enzymes, and enzyme stoichiometry (Figure 9a). Conversely, it was discovered that fungal α diversity strongly correlated with soil characteristics and enzyme activities. There was strong positive correlation between fungal species diversity and soil SOC and SOC:TP (*p* < 0.001). It was positively correlated with C-acquiring enzymes and P-acquiring enzymes (*p* < 0.01). Furthermore, fungi species richness was significantly positively correlated with soil pH, SWC, SOC, SOC:TN, and SOC:TP (*p* < 0.01) and significantly negatively correlated with EAA_C:P_ and EAA_N:P_ (*p* < 0.05).

Each environmental variable explained 52.71% of the variation in soil enzyme activity (Figure 9b.). The contributions of the first and second axis variables were 52.55% and 0.16%, respectively. Based on importance in explaining soil enzyme activity and their measurement ratio, the soil environmental factors were ranked as SOC:TP > SWC > SOC > TN > SOC:TN > pH > TP. This suggested that the key soil parameter influencing soil enzyme activity and measurement ratio was SOC:TP (*p* < 0.01).

## 4. Discussion

### 4.1. Changes in the Composition and Diversity of the Soil Microbial Community

Different land use types have changed the types of surface vegetation, affected the content of soil nutrients and thereby influencing the soil microbial community [33]. The dominant bacterial phyla for all four land use types in the northern mountains of Hebei were Proteobacteria, Actinobacteria, and Acidobacteriota, accounting for more than 50% of the total bacterial sequences. Wang et al. [34] reported similar results. Proteobacteria, which are primarily facultative or aerobic bacteria, exhibit extremely strong adaptability to the surrounding environment and can adapt to multiple ecosystems, which explains their high relative abundance in most land use types [35,36,37]. Proteobacteria in farmland was higher than that in the other three land use types. This may be because Proteobacteria are commonly found in agricultural soil. This outcome was consistent with what Angelo et al. [18] reported. Acidobacteria can grow rapidly in nutrient-deficient environments. Acidobacteria were most abundantly found in planted forest. This might be explained by the deficiency of nutrients and the acidic soil in planted forest, which resulted from planted forest being developed with human intervention. In contrast to this study, Chen et al. [38] found a higher abundance of Acidobacteria in farmland compared with other land use types, which may be due to geographical differences.

Ascomycota and Basidiomycota were the most prevalent fungal phyla at the ASV level in the investigation, which is in line with the findings of numerous other investigations. Soil Ascomycota and Basidiomycota are dominant fungi in the four land use types. Although there are differences in geographical location [39] and aboveground vegetation [40] among the four land use types in this study, the status of the dominant fungi remains unchanged. However, there are still differences in relative abundance and community composition between Ascomycota and Basidiomycota in terms of land use types. For example, studies of soil microorganisms in different land use types in northern mountainous areas have found that the relative abundance of Ascomycota is highest in shrublands, farmlands, and grassland, while the proportion of the relative abundance of Basidiomycota is the highest in forests [41]. This result was consistent with the horizontal distribution of fungal phyla seen in the Pearl River Estuary under various land use scenarios [42].

Microbial α diversity analysis revealed that the microbial (bacterial and fungal) diversity and richness in farmland were lower than those for other land use types. According to certain research, habitat variations may contribute to a decrease in soil microbial diversity, particularly when human activities like tillage, fertilization, and other actions are involved [43]. Nevertheless, other research has revealed the opposite outcome, showing an increase in root exudates and crop residues in soil after fertilization or crop rotation, which further increased the microbial diversity and richness of soil [44]. The farmland in this study adopted a rotation tillage pattern of corn–sunflower–potato, which might be more conducive to an increase in soil microbial diversity compared with the common continuous tillage pattern [45]. Meanwhile, this study found that the soil nutrient content of farmland was the lowest among the four land use types, and the nutrient supply capacity was relatively weak. However, compared with grassland, secondary forest, and planted forest, farmland is affected by human activities, which interferes with its soil structure and nutrient cycling process. The content of organic carbon and total nitrogen in farmland soil is low. Therefore, the microbial diversity of farmland soil in this area is lower than that of other land use types. In forest and grassland areas, the low diversity of soil microorganisms in farmland will have certain impacts on agricultural production in this region, including crop yields, etc. A comparative experiment should be set up to further verify the role of soil microbial diversity in agricultural production in this area.

Overall, the soil’s depth had an impact on the microbial community’s structure. One important environmental factor influencing the richness and complexity of the soil microbial community is the depth of the soil layer [46,47]. Based on the findings of this study, the abundance of some bacterial phyla (like Proteobacteria) declined as soil depth increased. This study found that soil nutrients generally decrease with increasing soil layer depth. Therefore, the energy available for soil bacteria to utilize decreases, and the relative abundance of microorganisms also decreases accordingly [48]. In an earlier investigation, the overall relative abundance of some microbial groups, such as Firmicutes, Chlorendella, and Ascomobacteria, increased with increasing soil depth, reflecting an increase in dominant microbiomes that can maintain the soil nutrient cycle and microbial metabolism with low energy availability [49]. Certain soil microorganisms may find it more difficult to survive in the underlying soil layer due to the filtering of vertical space between soil layers, which further lowers the diversity of soil microbes [50]. However, unlike the other three land use types, the diversity and abundance of bacteria in the 0–10 cm layer of farmland soil are significantly lower than those in the 20–50 cm layer, which is different from the results obtained by most studies. As farmland is a “non-steady-state” environment, it can be affected by human disturbances (such as fertilization and ploughing). The farmland in the study area is often ploughed, which may be one of the reasons for the unstable surface soil conditions of the farmland, which is not conducive to the growth of soil microorganisms and leads to a reduction in bacterial diversity and abundance.

### 4.2. Differences in Soil Microbial Metabolism Limitations

Aboveground and underground bio-communities, soil properties, and input and output of soil organic matter directly affect soil enzyme activity. In this study, enzyme activity was lowest in farmland. This could be because of the low organic matter content, vegetation litter, and root secretion, which lead to low secretion and activities of soil extracellular enzymes [51]. It may also be related to the farming measures adopted in farmland [52]. The soil nutrient content also reflects microbial enzyme activity, and microbial enzyme activity is different for different land use types. Furthermore, forest also showed a close relationship between soil organic carbon (SOC) and total nitrogen (TN). According to studies, NAG is the primary enzyme associated with soil N and is necessary for the circulation of nutrients [53]. Soil microbes may have access to more substrates and energy due to the high SOC and N content of forest soil [54]. Meanwhile, forest vegetation litter and root secretions further stimulate the secretion of extracellular enzymes, thus increasing soil enzyme activity.

The ecological stoichiometric ratio of soil enzyme activity was determined by log-transforming the data, and it was found to be 1:1.46:0.73. This ratio deviates from the global ecosystem soil enzyme stoichiometric ratio of 1:1:1 [6]. This suggested that different land use types had high activity of N-acquiring enzymes, reflecting a relative lack of nitrogen in this region. Most of the land use types had a vector angle of <45°, which indicated nitrogen limitation. Analysis of soil enzyme vector length revealed that there was no discernible variation in carbon restriction among the four land use types. Furthermore, the degree of carbon restriction in secondary forest and planted forest was lower than that in farmland and grassland. Similar findings from other studies have suggested that a higher SOC content in the soil can supply more carbon sources for microbial growth, resulting in weaker carbon limitation [55].

The activities of C-, N-, and P-acquiring enzymes generally decline with increasing soil depth, which aligns with the decreasing trend of soil nutrients with depth. The surface soil’s humus and organic matter accumulation is enhanced by prolonged deposition and breakdown of highly polymerized plant residues, altering the soil’s structure [56]. Deep soil has less enzyme activity than surface soil because it cannot supply microbial enzymes with enough substrates and energy, due to its lower nutrient content [57]. Soil enzyme stoichiometric ratio analysis showed that deep soil was more severely limited by N than top soil. The soil vector angles further confirmed higher N limitation in deep farmland soil compared to surface soil. In contrast, deep soil from secondary forest and plantations, and in grassland soil, had less N limitation than the respective surface soil, with increased P limitation. This could be because, as soil depth increases, the distribution of planted vegetation’s roots decreases relatively. Deep-rooted plant roots in secondary forest and planted forest compete with soil microorganisms for phosphorus, resulting in reduced nitrogen limitation and increased P limitation [58]. In a study by Liu et al. [25] on the nutrient restriction of forest soil in China, it was also found that deep-rooted plants and soil microorganisms compete for nutrient elements, emphasizing their competition for phosphorus. The phosphorus restriction of soil microorganisms increases with increasing soil layer depth, which is consistent with the results obtained in this study. The vector length of enzymes reflects potential hydrolysis of organic carbon in the soil, but it cannot measure carbon restriction in soil microorganisms [59]. Because the carbon content in deep soil is lower than that in the surface layer, which inhibits the growth and metabolism of microorganisms, the medium vector length for secondary forests and planted forests decreases with increasing soil depth [58].

### 4.3. Key Factors Affecting the Composition and Metabolic Limitation of Soil Microbial Communities

The soil microbial bacterial community composition was mainly influenced by SOC, TN, and SOC:total phosphorus (TP), while the fungal community composition was primarily affected by soil water content (SWC) and TN:TP. These results concurred with those of an earlier study, reporting SOC and TN as key factors affecting soil bacterial communities [60]. Yu et al. [61] studied carbon fixation and microbial regulation mechanisms in grassland soil and observed that the fungal community was more affected by SWC. The environmental parameters influencing fungal diversity in the current study were comparable to those described by Yang et al. [62], who found that soil pH, bulk density, and SOC concentration were the necessary soil factors determining soil microbial diversity. Contrary to the findings of the majority of previous investigations, soil bacterial populations were less impacted by environmental influences. For example, Yan et al. [63] observed that environmental factors were closely related to soil bacterial diversity. This may be due to the heterogeneity of soils in different habitats.

The bioavailability of soil nutrients is mainly based on the microbial community and the environmental conditions in soil [64]. In this study, N limitation of soil microorganisms was found to be related to SOC:TP, SWC, and SOC. Since soil microorganisms are N-limited, they try to obtain as much N as possible from the environment, which leads to higher activity of N-acquiring enzymes. SOC has an important relationship with soil microbial nitrogen limitation. Soils with a high SOC level are better for microbial development and enzyme synthesis because they have a stronger water-holding capacity and a higher effective carbon content [65]. This explains the positive correlations between N-acquiring enzymes and SOC. In this study, N limitation in plantation areas was more severe than that in farmland, grassland, and secondary forest. Due to the higher SOC content in plantation soil, more N-acquiring enzymes are released by microbes to alleviate the N limitation [23]. These plants then compete with microorganisms for N, thus aggravating N limitation in soil. Yuan et al. [66] conducted nitrogen addition experiments in semi-arid grasslands in northern China and found that there is a potential competitive relationship between soil microorganisms and plants, and this competition may be related to soil nitrogen limitation. In addition, plant roots and microorganisms have a certain degree of spatial overlap, and both are significantly related to inorganic nitrogen in the soil. The root systems of plants absorb nitrogen at a faster rate than microorganisms [67].

Cregger et al. [68] found that the increase in SWC is conducive to the growth of plants. Changes in SWC were shown to be negatively connected with carrier length and carrier angle and favorably correlated with enzyme activity in this investigation. SOC:TP was the most significant factor affecting nutrient metabolic limitation in soil microorganisms. Research has demonstrated that the microbial enzyme stoichiometry ratio and enzyme activity in soil can be significantly impacted by the stoichiometric ratio of soil nutrients [69,70]. In the study, the SOC:TP content of the four land use modes was higher, which was closely related to the activity and stoichiometric ratio of the three extracellular enzymes. These findings proved the significant relationships between SOC:TP and microbial nutrient limitation.

## 5. Conclusions

In this study, changes in soil microbial diversity and metabolic limitation with soil layer depth were investigated in agricultural farmland, grassland, secondary forest, and planted forest in the mountainous forest-grass ecotone of northern China. In summary, the microbial diversity of agricultural land was significantly lower compared to that of other land uses. Soil microorganisms were N-limited regardless of soil texture and depth. Among the four land uses, planted forest exhibited the most severe N limitation. Notably, N limitation in farmland increased with soil depth, whereas grassland, secondary forest, and planted forest showed the opposite trend. This research shows that SOC, TN, and SOC:TP affected bacterial community composition, while SWC, SOC, and TN:TP affected fungal community composition. SOC:TP is closely related to soil microbial nitrogen limitation. This study emphasized that land use types are more influential for soil microbial community than soil depth, and that soil nutrients and their stoichiometric ratios play a key role in microbial metabolic limitation. Land use changes the input of soil nutrients by aboveground plants, which affects the physical and chemical properties of soil, microbial community diversity, and microbial metabolic limitation. The vertical filtration effect between soil layers reduces soil nutrients, making the microbial diversity and enzyme activity of surface soil greater than that of deep soil. In the future, more attention should be paid to the action mechanisms of soil microorganisms in different land use types within the ecologically fragile area of interlaced forests and grasslands, providing a basis for more precisely optimizing land use management strategies. Overall, this study advances the understanding of soil microbial functions under different land use types in the forest-grass ecotone of northern China and emphasizes the need to incorporate vertical changes in microbial properties along the soil layer in future studies to predict the dynamics of biogeochemical cycles within ecosystems in this ecologically critical zone in the context of climate warming.

## Figures and Tables

**Table 1:** Basic overview of the sample plot.

Land Use Types	Elevation (m)	Mean BDH (cm)	Mean Tree Height (m)	Crown Density	Cover Degree %
Farmland	1357	/	/	/	80
Grassland	1375	/	/	/	77
Secondary forest	1402	14.44	11.2	0.70	/
Planted forest	1406	13.75	10.8	0.53	/

## Data Availability

The data presented in this study are available on request from the corresponding author. The data are not publicly available due to privacy restrictions.

## Abbreviations

SWC	Soil Water Content
SOC	Soil Organic Carbon
TN	Total Nitrogen
TP	Total Phosphorus
